# Effects of sex, age, choice of surgical orthodontic treatment, and skeletal pattern on the psychological assessments of orthodontic patients

**DOI:** 10.1038/s41598-022-12129-0

**Published:** 2022-06-01

**Authors:** Sayaka Hino, Aya Maeda-Iino, Takakazu Yagi, Shoko Nakagawa, Shouichi Miyawaki

**Affiliations:** 1 Department of Orthodontics and Dentofacial Orthopedics, Field of Developmental Medicine, Health Research Course, Graduate School of Medical and Dental Sciences, Kagoshima University, 8-35-1 Sakuragaoka, Kagoshima, 890-8544 Japan; 2 Department of Oral Health, Faculty of Health Sciences, Kobe Tokiwa University, Hyogo, Japan; 3 Department of Orthodontics, Center of Developmental Dentistry, Kagoshima University Hospital, Kagoshima, Japan

**Keywords:** Psychology, Medical research

## Abstract

We aimed to examine the effects of sex, age, choice of surgical orthodontic treatment, and skeletal pattern on psychological assessment scores of orthodontic patients before edgewise treatment. They completed the State-Trait Anxiety Inventory (STAI), Beck Depression Inventory (BDI)-II, and the psychological domain of the World Health Organization Quality of Life 26 (Psych-QOL) for assessment of anxiety, depression, and body image, respectively. No significant effects on psychological assessment scores due to sex or age differences were found. Surgical orthodontic treatment patients and patients with skeletal Class III had significantly higher STAI-Trait and/or BDI-II scores and lower Psych-QOL score. Based on the linear mixed-effects model, the choice of surgical orthodontic treatment had a significant effect on the STAI-Trait, BDI-II, and Psych-QOL scores. No significant interaction effect was found between the choice of surgical orthodontic treatment and the skeletal pattern by ANB angle. Patients with skeletal Class I or III who chose surgical orthodontic treatment had higher STAI-Trait and/or BDI-II scores and/or lower body image score, respectively. These results suggest that patients who chose surgical orthodontic treatment, particularly those with skeletal Class I and III, may be more prone to experience anxiety and depression and have body image dissatisfaction.

## Introduction

Different malocclusions and orofacial morphologies have been associated with oral health and function^1–8^. Visible malocclusions are closely related to psychological problems; thus, it is important for orthodontists to be familiar with the psychological tendencies associated with different types of malocclusions. Among the various types of malocclusions, those requiring surgical orthodontic treatment have been associated with many psychological problems^3–8^. Similarly, when the pre-treatment psychological profiles of patients referred for orthognathic surgery were compared with those of patients who were scheduled to receive non-orthognathic surgery, the patients referred for orthognathic surgery had higher levels of anxiety, higher numbers of individuals in their social support network, and lower body and facial body image scores^3,6^. Skeletal Class III patients are especially well-known to have more psychological problems than skeletal Class I or II patients^4,5,7^.

On the other hand, sex and age are related to psychological problems and quality of life (QOL)^9^. Female patients requiring orthognathic surgical treatment were more aware of their facial deformities and reported a greater social detriment than their male counterparts^3,6^. In addition, female patients and younger patients (12–19 years) tended to have higher scores for oral health-related quality of life (OHRQoL) than male patients and older patients (20–35 years), respectively^2^. Thus, evaluations of psychological problems and QOL of orthodontic patients should take into account the degree or classification of malocclusion, sex, and age. However, the relationship of psychological problems or QOL with factors such as sex, age, choice of surgical orthodontic treatment, and skeletal pattern has not been clarified to date.

Therefore, this study aimed to examine the effects of sex, age, choice of surgical orthodontic treatment, and skeletal pattern on the anxiety, depression, and psychological domains of QOL in orthodontic patients before edgewise treatment.

## Materials and methods

### Participants

**Figure 1:**
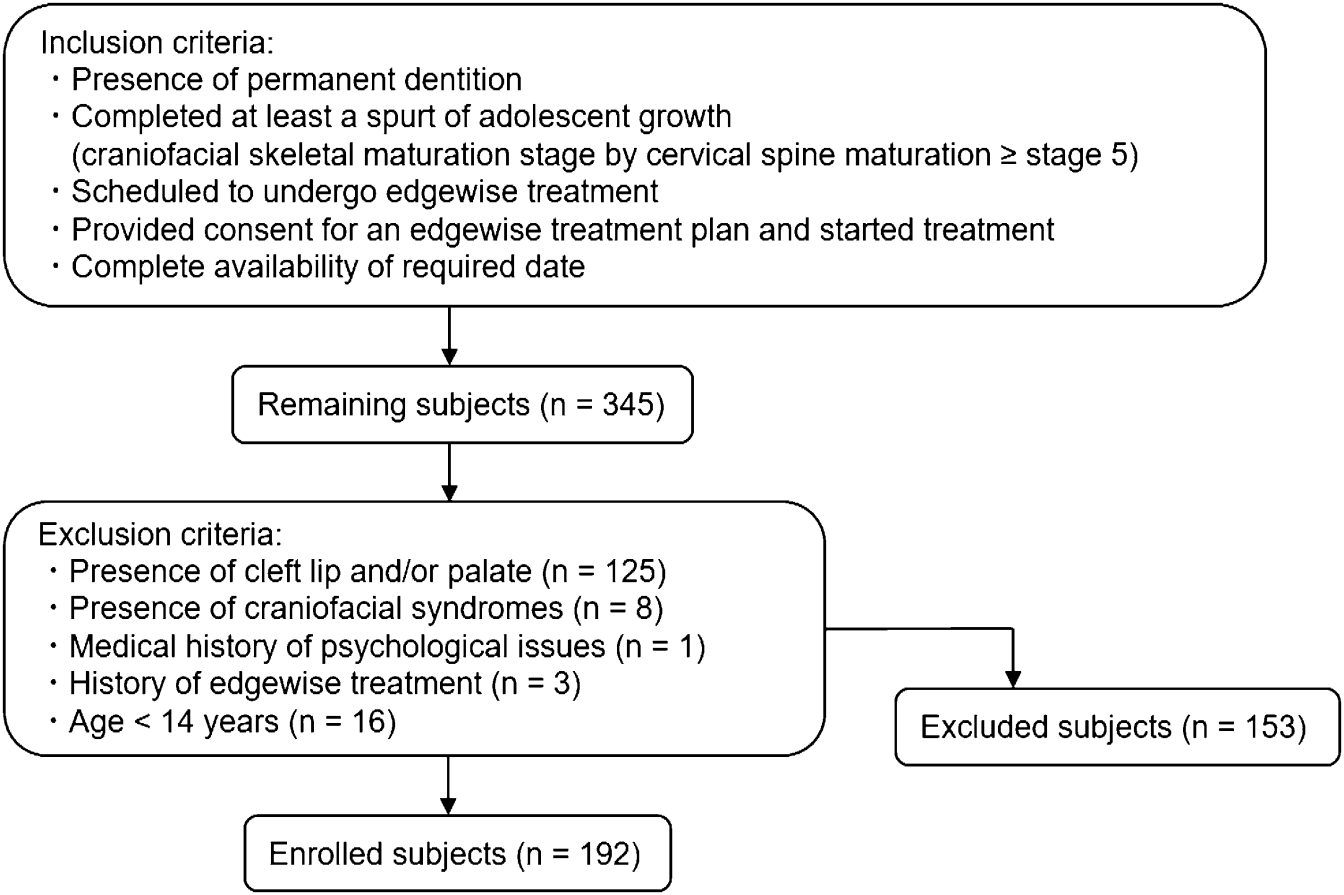
Flowchart of participant enrollment.

Study participants included consecutively selected patients who received diagnoses on the basis of panoramic and cephalometric radiographs, dental casts, and psychological tests before edgewise treatment at the Department of Orthodontics at Kagoshima University Hospital, Kagoshima, Japan, from 2013 to 2021. Among these consecutive patients, those who met the inclusion and exclusion criteria were analyzed in this study (*n* = 192) (Fig. 1). The participants who had been treated for psychological problems, including depression and/or anxiety, were excluded from this study (*n* = 1). The study design was approved by the Kagoshima University Ethics Committee (approval number #200310(661)-1), and the need for written informed consent was waived in the retrospective analysis. Instead, we applied the optout method to obtain consent for this study via the web sites of Kagoshima University Hospital according to the guidelines. On the web pages, it was announced that the records collected for orthodontic treatment in patients had been anonymized, then used in the study. Patients (and their parent(s) if they were under the age of 20) were given the opportunity to refuse to participate in this study, however none of them refused to participate in the study. The study was conducted according to the ethical principles outlined in the Declaration of Helsinki.

The participants were classified by sex (male or female) and age (adolescents: 14 ≤ age < 20 years; adults: age ≥ 20 years). Patients diagnosed without jaw deformity by an orthodontist were treated with orthodontic treatment only. Among the patients diagnosed with jaw deformity by an orthodontist and oral surgeon, those with severe jaw deformity who could not expect improvement in occlusion by orthodontic treatment only were treated with surgical orthodontic treatment. If a patient had mild jaw deformity and orthodontic treatment alone could improve the occlusion, either treatment with orthodontic treatment alone or surgical orthodontic treatment were proposed, and the treatment adopted was at the choice of the patient. In both orthodontic treatment methods, adolescent patients selected the treatment with their parent(s). Those who did not receive surgical orthodontic treatment were defined as “non-surgical orthodontic treatment patients” (NONS group; *n* = 143), whereas those who were diagnosed as showing jaw deformity and chose surgical orthodontic treatment were defined as “surgical orthodontic treatment patients” (SURG group; *n* = 49).

### Evaluation of skeletal morphology using lateral and frontal cephalometric radiographs

**Figure 2:**
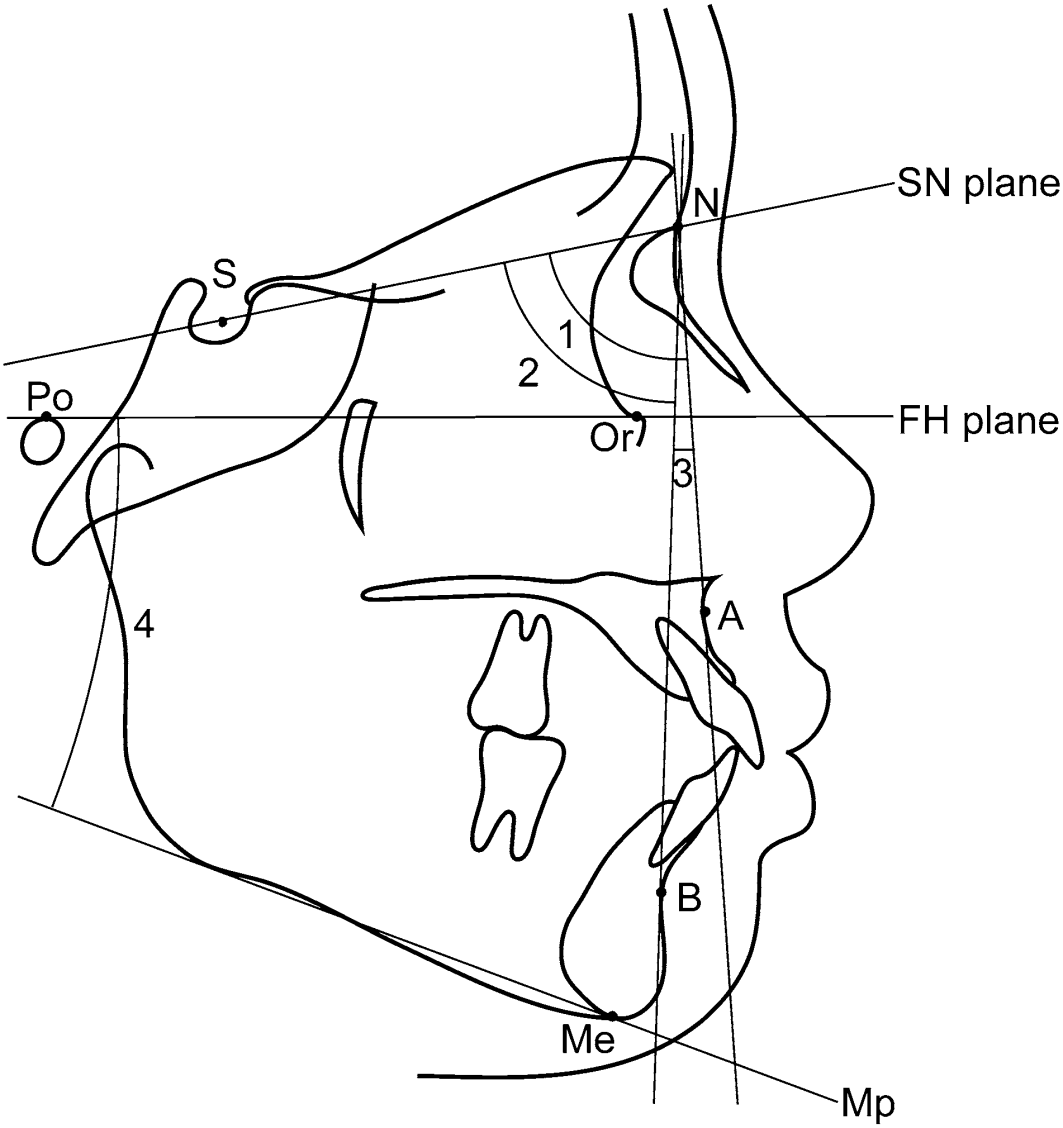
Lateral cephalometric analysis. 1, SNA angle (angle between the SN plane and NA line); 2, SNB angle (angle between the SN plane and NB line); 3, ANB angle (angle between the NA line and NB line); 4, Mandibular plane (Mp)—FH angle (angle between Mp and FH plane).

**Figure 3:**
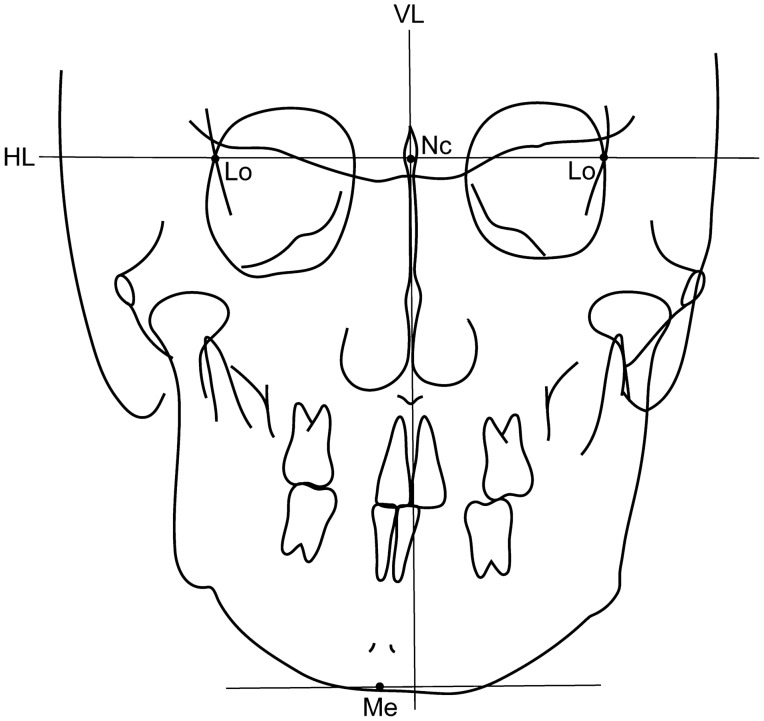
Frontal cephalometric analysis. *VL* vertical line, *HL* horizontal line.

Lateral cephalometric radiographs were used to evaluate anterior–posterior or vertical skeletal morphology (Fig. 2). Measurements on the lateral cephalometric radiographs were determined using the WinCeph 9.0 software (Compudent, Koblenz, Germany). To assess the anterior–posterior (skeletal Class I, II, or III) and vertical (average, low, or high angle) skeletal patterns, the participants were classified based on ANB and FMA angles, respectively, and divided into three groups based on the Japanese normal mean value ± 1 SD of each cephalometric measurement^10^. The frontal cephalometric radiographs of all patients were evaluated to determine mandibular asymmetry (Fig. 3). The horizontal distance between the vertical line (VL) and menton (Me) was measured as the deviation of the mandible^11^. If the distance between the VL and Me exceeded 4 mm, the patient was considered to have mandibular asymmetry^5^.

To assess the intra-examiner reproducibility and reliability of the measurements, 20 randomly selected cephalometric radiographs were retraced after a minimum interval of 2 months. Evaluation of discrepancies in measurement between the original and retraced radiographs (matched paired *t*-test) revealed no statistically significant differences.

### Evaluation of psychological state

Psychological testing of patients was performed before edgewise treatment. Patients completed the State-Trait Anxiety Inventory (STAI)^12,13^, the Beck Depression Inventory (BDI)-II^14^, and the World Health Organization Quality of Life 26 (WHO-QOL26)^15^ for assessment of anxiety, depression, and QOL levels, respectively. The Japanese version of these psychological evaluations was used^12–15^. All questions were administered as a self-completed questionnaire, and the total score for each test was used for statistical analysis. To avoid bias resulting from others’ influence, each patient completed the questionnaires alone in a private room. Internal consistencies for these scales were evaluated with Cronbach’s alpha coefficient.

### STAI

The anxiety status was measured by the STAI^12^, a self-reported instrument that consists of separate self-reported scales measuring two distinct anxiety concepts: state anxiety (how the participants felt “at this moment”) and trait anxiety (how the participants “generally” felt). We used the Japanese version of STAI^12^, which has been widely used and validated in different populations and has also been frequently used in orthodontic patients^13^. Trait anxiety (STAI-Trait) was evaluated in this study. The scale consists of 20 statements, which are evaluated using a four-point Likert scale ranging from 1 to 4. For example: “I feel pleasant” describes asking about the participants’ underlying anxiety. Participants answer on a scale form: (1, Indicates rarely; 2, Sometimes; 3, Often; and 4, Almost always). Higher scores reflect higher anxiety. The STAI-Trait scores ranged between 20 and 80 for each subscale. For the total scale, Cronbach’s alpha was 0.89.

### BDI-II

The BDI-II Japanese version^14^ was used to assess depression. The BDI-II covers a wide range of items from sadness and a sense of failure to self-dislike, social withdrawal, and indecisiveness. It consists of 21 items that are presented in multiple-choice form and is designed to assess the severity of depression symptoms in the last two weeks. Patients selected responses that best suited their current situation to determine the intensity/severity of depression. The scores ranged from 0 to 3 for each item. In the item “Unhappiness,” participants answer on a scale form: (0, I do not feel unhappy; 1, I feel unhappy; 2, I am unhappy; and 3, I am so unhappy that I can’t stand it). In the BDI-II, a higher score indicated a greater number of symptoms of depression. The total scores ranged from 0 to 63. For the total scale, Cronbach’s alpha was 0.88.

### WHO-QOL26

To assess the participants’ quality of life, happiness, and mental health before orthodontic treatment, we administered the WHO-QOL26, a 26-item self-reported measure designed to assess QOL. Twenty-four items measured the four domains of QOL–physical, psychological, social, and environmental–and the other two items measured overall QOL and general health. In this study, we used the Japanese version of the WHO-QOL26^15^ and analyzed the results only for the psychological domain of the WHO-QOL26 (Psych-QOL). For example: “How much do you enjoy life?” asks how much participants have experienced certain things in the last two weeks. Participants answer on a scale form: (1, Not at all; 2, A little; 3, A moderate amount; 4, Very much; and 5, An extreme amount). The score for each question ranges from 1 to 5 with higher scores reflecting higher QOL. The mean score was used for the analysis. For the total scale, Cronbach’s alpha was 0.78.

### Statistical analysis

Each outcome variable was assessed for normality, as this distributional property was required prior to undertaking analysis. The overall scores of STAI-Trait, BDI-II, and Psych-QOL didn’t show a normal distribution. Thus, intergroup differences in the distributed variables (sex, age, choice of surgical orthodontic treatment, and skeletal pattern) were evaluated using the Mann–Whitney *U* test or Kruskal–Wallis test as nonparametric tests. Statistical significance was set at *P* < 0.05, after Bonferroni adjustment for multiple comparisons. For categorical variables (choice of surgical orthodontic treatment and anterior–posterior skeletal pattern), significance was determined using Fisher’s exact test (two-group comparisons). To examine the main and interaction effects of group (choice of surgical orthodontic treatment and anterior–posterior skeletal pattern) on the STAI-Trait, BDI-II, and Psych-QOL scores, we used the generalized linear model (GLM). However, Box’s M showed that the criteria for equality of covariance matrices were not met. In addition, Levene’s test for equality of variance of BDI-II score revealed violated assumptions of homogeneity and missing data of psychological evaluation values averaged 4.9% of the total. To resolve those problems, the linear mixed-effects models (LMM), which are robust to unbalanced designs and are able to handle incomplete participants data, were used. LMM used a full information maximum likelihood approach to missing data, enabling us to analyze the most accurate estimation of effects and unbiased results. All statistical analyses were performed using SPSS version 28.0 for Windows (IBM Corporation, Armonk, NY, USA). The sample size was calculated using a conventional alpha level of 0.05 and a power level of 0.8. Fisher’s exact test and Mann–Whitney *U* test required at least 29 and 27 participants for each group (effect size: 0.8), respectively. The Kruskal–Wallis test and LMM required at least 159 participants, respectively (both effect size: 0.25). If the sample size was lower than the limit, we performed post-hoc power calculations (1-β).

### Ethical considerations

The study design was approved by the Kagoshima University Ethics Committee (approval number #200310(661)-1). The study was conducted according to the ethical principles outlined in the Declaration of Helsinki.

## Results

Comparison of the scores for psychological states in each group showed no significant difference in STAI-Trait, BDI-II, and Psych-QOL scores in relation to sex, age, vertical skeletal pattern, and mandibular symmetry/asymmetry (Tables 1 and 2). The SURG group showed significantly higher STAI-Trait (*P* < 0.001) and BDI-II (*P* = 0.004) scores and lower Psych-QOL score (*P* < 0.001) than the NONS group. The STAI-Trait and BDI-II scores of patients with skeletal Class III were significantly higher than those with skeletal Class I and II (*P* < 0.05). The Psych-QOL score of patients with skeletal Class III was significantly lower than that of patients with skeletal Class I and II (*P* = 0.022).

**Table 1:** Comparison of psychological assessments between groups classified by sex, age, and choice of surgical orthodontic treatment.

Psychological assessment items	Sex						
	Male (n = 62)			Female (n = 130)			*P*^a^
	Mean	SD	Median	Mean	SD	Median	
STAI-Trait	40.37	9.70	40.50	40.97	9.06	40.00	0.687
BDI-II	5.89	5.34	4.00	7.03	6.63	5.00	0.435
Psych-QOL	3.56	0.68	3.50	3.38	0.59	3.33	0.077

Psychological assessment items	Age						
	Adolescent (n = 87)			Adult (n = 105)			*P*^a^
	Mean	SD	Median	Mean	SD	Median	
STAI-Trait	39.92	8.55	40.00	41.48	9.79	41.00	0.260
BDI-II	6.08	5.63	4.00	7.14	6.71	5.00	0.312
Psych-QOL	3.54	0.58	3.50	3.36	0.65	3.33	0.147

Psychological assessment items	Choice of surgical orthodontic treatment						
	NONS (n = 143)			SURG (n = 49)			*P*^a^
	Mean	SD	Median	Mean	SD	Median	
STAI-Trait	39.08	8.57	38.00	45.77	9.49	46.00	< 0.001***
BDI-II	5.70	5.15	4.00	9.47	8.15	6.00	0.004**
Psych-QOL	3.57	0.58	3.50	3.07	0.59	3.00	< 0.001***

*NONS* non-surgical orthodontic treatment, *SURG* surgical orthodontic treatment, *STAI* State-Trait Anxiety Inventory, *BDI-II* Beck Depression Inventory-II, *Psych-QOL* psychological domain of the World Health Organization Quality of Life 26, *SD* standard deviation.

^a^Mann–Whitney *U* test.

**P < 0.01; ***P < 0.001.

**Table 2:** Comparison of psychological assessments between groups classified by maxillofacial morphology.

Psychological assessment items	Anterior–posterior skeletal pattern^a^												
	Skeletal Class I (n = 84)			Skeletal Class II (n = 45)			Skeletal Class III (n = 63)			*P*^c^	Skeletal Class		
	Mean	SD	Median	Mean	SD	Median	Mean	SD	Median		I vs. II	II vs. III	I vs. III
STAI-Trait	40.28	9.86	40.00	37.93	8.69	37.00	43.50	8.15	43.50	0.005**	0.485	0.004**	0.088
BDI-II	6.79	5.74	6.00	4.47	4.33	3.00	8.06	7.59	5.00	0.026*	0.096	0.027*	1.000
Psych-QOL	3.51	0.64	3.50	3.57	0.62	3.58	3.26	0.58	3.17	0.022*	0.100	0.032*	1.000

Psychological assessment items	Vertical skeletal pattern^b^												
	Average angle (n = 117)			Low angle (n = 43)			High angle (n = 32)			*P*^c^			
	Mean	SD	Median	Mean	SD	Median	Mean	SD	Median				
STAI-Trait	39.81	9.08	40.00	42.28	8.26	41.00	42.22	10.85	41.50	0.214			
BDI-II	6.26	6.09	4.00	7.72	6.80	6.00	6.69	6.09	4.50	0.530			
Psych-QOL	3.44	0.65	3.33	3.44	0.61	3.50	3.43	0.57	3.50	0.994			

Psychological assessment items	Mandibular symmetry/asymmetry												
	Symmetry (n = 140)						Asymmetry (n = 52)						*P*^d^
	Mean		SD		Median		Mean		SD		Median		
STAI-Trait	40.37		9.10		40.00		41.85		9.66		41.00		0.480
BDI-II	6.83		6.01		5.00		6.21		6.90		3.50		0.186
Psych-QOL	3.44		0.62		3.42		3.44		0.64		3.50		0.803

*STAI* State-Trait Anxiety Inventory, *BDI-II* Beck Depression Inventory-II, *Psych-QOL* psychological domain of the World Health Organization Quality of Life 26, *SD* standard deviation.

^a^Classified by ANB angle as follows: skeletal Class I (Japanese normal mean value - 1 SD ≤ ANB angle ≤ Japanese normal mean value + 1 SD), Class II (ANB angle > Japanese normal mean value + 1 SD), and Class III (ANB angle < Japanese normal mean value - 1 SD).

^b^Classified by FMA angle as follows: average angle (Japanese normal mean value - 1 SD ≤ value of FMA angle ≤ Japanese normal mean value + 1 SD), low angle (value of FMA angle < Japanese normal mean value - 1 SD), and high angle (value of FMA angle > Japanese normal mean value + 1 SD).

^c^Kruskal–Wallis test.

^d^Mann–Whitney *U* test.

*P < 0.05; ** *P* < 0.01.

We used LMM and evaluated the effects of choice of surgical orthodontic treatment and differences in skeletal pattern based on the ANB angle on each psychological state score. The choice of surgical orthodontic treatment (NONS or SURG groups) as the main effect significantly affected the STAI-Trait (*F* (1, 190) = 12.995, *P* < 0.001), BDI-II (*F* (1, 192) = 8.385, *P* = 0.004), and Psych-QOL scores (*F* (1, 166) = 18.802, *P* < 0.001; Table 3). However, the skeletal pattern by ANB angle as the main effect significantly didn’t affect STAI-Trait, BDI-II, and Psych-QOL scores. No significant interaction effect was found between the choice of surgical orthodontic treatment and the skeletal pattern by ANB angle (Table 3). The values of each anterior–posterior skeletal pattern group were compared between the two groups of surgical orthodontic treatment (i.e., NONS and SURG) (Table 4).

**Table 3:** Results of linear mixed-effects model of psychological evaluation scores.

Psychological assessment items	Main effect				Interaction	
	Choice of surgical orthodontic treatment (NONS/SURG)		ANB angle (Skeletal Class I/II/III^a^)		Choice of surgical orthodontic treatment × ANB angle	
	*F*	*P*^*b*^	*F*	*P*^*b*^	*F*	*P*^*b*^
STAI-Trait	12.995	< 0.001***	1.251	0.289	1.252	0.288
BDI-II	8.385	0.004**	2.562	0.080	1.071	0.345
Psych-QOL	18.802	< 0.001***	0.043	0.958	1.144	0.321

*NONS* non-surgical orthodontic treatment, *SURG* surgical orthodontic treatment, *ANB angle* anterior–posterior skeletal pattern classification by ANB angle, *STAI* State-Trait Anxiety Inventory, *BDI-II* Beck Depression Inventory-II, *Psych-QOL* psychological domain of the World Health Organization Quality of Life 26.

^a^Skeletal Class I (Japanese normal mean value - 1 SD ≤ ANB angle ≤ Japanese normal mean value + 1 SD), Class II (ANB angle > Japanese normal mean value + 1 SD), and Class III (ANB angle < Japanese normal mean value - 1 SD).

^b^Linear mixed-effects model.

** *P* < 0.01; *** *P* < 0.001.

**Table 4:** Comparison of psychological assessments between groups classified by maxillofacial morphology^a^ and choice of surgical orthodontic treatment.

Variables	Skeletal Class I									
	NONS (n = 76)			SURG (n = 8)			*P*	1-β		
	n/n, Mean	SD	Median	n/n, Mean	SD	Median				
Sex; male/female	31/45			3/5			1.000^b^	0.04		
Age (y)	22.07	7.64	20.00	25.16	12.42	19.88	0.578^c^	0.12		
SNA angle (°)	82.18	3.64	81.80	81.41	3.29	81.55	0.637^c^	0.09		
SNB angle (°)	78.46	3.89	78.00	77.94	3.01	77.70	0.552^c^	0.07		
ANB angle (°)	3.60	1.03	3.50	3.48	1.49	3.80	0.994^c^	0.06		
FMA angle (°)	26.54	5.71	26.65	36.59	3.64	36.55	< 0.001***^c^	1.00		
Me deviation (mm)	2.71	2.76	1.83	4.82	3.21	4.91	0.049*^c^	0.45		
STAI-Trait	39.29	9.42	38.00	49.50	9.71	49.50	0.012*^c^	0.79		
BDI-II	6.18	5.33	5.00	12.50	6.63	12.50	0.007**^c^	0.78		
Psych-QOL	3.57	0.62	3.50	2.93	0.58	3.00	0.010*^c^	0.79		

Variables	Skeletal Class II									
	NONS (n = 40)			SURG (n = 5)			*P*	1-β		
	n/n, Mean	SD	Median	n/n, Mean	SD	Median				
Sex; male/female	7/33			2/3			0.258^b^	0.01		
Age (y)	22.78	7.15	20.42	27.62	4.27	25.33	0.042*^c^	0.38		
SNA angle (°)	83.32	3.55	83.30	82.60	3.82	80.80	0.687^c^	0.07		
SNB angle (°)	75.97	3.51	76.05	74.24	4.47	72.80	0.428^c^	0.14		
ANB angle (°)	7.35	1.73	6.90	8.36	1.09	8.00	0.080^c^	0.29		
FMA angle (°)	30.44	5.84	30.75	31.18	12.10	33.10	0.428^c^	0.05		
Me deviation (mm)	3.04	3.05	1.95	1.74	1.04	1.60	0.687^c^	0.06		
STAI-Trait	37.25	7.37	36.50	43.40	16.09	47.00	0.280^c^	0.17		
BDI-II	4.22	3.91	3.00	6.40	7.23	5.00	0.739^c^	0.12		
Psych-QOL	3.66	0.51	3.67	2.93	0.94	2.50	0.146^c^	0.49		

Variables	Skeletal Class III									
	NONS (n = 27)			SURG (n = 36)			*P*			
	n/n, Mean	SD	Median	n/n, Mean	SD	Median				
Sex; male/female	8/19			11/25			1.000^b^			
Age (y)	24.71	8.54	22.08	21.63	7.49	19.46	0.063^c^			
SNA angle (°)	80.96	4.01	81.50	81.92	3.56	82.15	0.235^c^			
SNB angle (°)	81.46	4.62	80.70	85.48	4.22	86.25	< 0.001***^c^			
ANB angle (°)	-0.51	1.27	-0.40	-3.55	2.62	-3.50	< 0.001***^c^			
FMA angle (°)	24.40	5.51	23.20	25.52	6.95	26.10	0.173^c^			
Me deviation (mm)	4.47	4.32	2.35	2.78	2.49	2.12	0.317^c^			
STAI-Trait	41.22	7.38	41.00	45.26	8.38	45.00	0.071^c^			
BDI-II	6.52	5.91	5.00	9.22	8.54	5.50	0.259^c^			
Psych-QOL	3.45	0.59	3.33	3.12	0.55	3.17	0.047*^c^			

Variables	NONS					SURG				
	*P*	1-β	Skeletal Class			*P*	1-β	Skeletal Class		
			I vs. II	II vs. III	I vs. III			I vs. II	II vs. III	I vs. III
Sex; male/female										
Age (y)	0.197^d^					0.067^d^				
SNA angle (°)	0.044*^d^	1.000	0.318	0.551	0.041*	0.944^d^				
SNB angle (°)	< 0.001***^d^	1.000	0.004**	0.021*	< 0.001***	< 0.001***^d^	1.000	1.000	0.001**	0.001**
ANB angle (°)	< 0.001***^d^	1.000	< 0.001***	< 0.001***	< 0.001***	< 0.001***^d^	1.000	1.000	< 0.001***	< 0.001***
FMA angle (°)	< 0.001***^d^	1.000	0.258	0.002**	< 0.001***	< 0.001***^d^	1.000	0.766	< 0.001***	0.189
Me deviation (mm)	0.337^d^					0.129^d^				
STAI-Trait	0.149^d^					0.534^d^				
BDI-II	0.177^d^					0.271^d^				
Psych-QOL	0.290^d^					0.612^d^				

*NONS* non-surgical orthodontic treatment, *SURG* surgical orthodontic treatment, *STAI* State-Trait Anxiety Inventory, *BDI-II* Beck Depression Inventory-II, *Psych-QOL* psychological domain of the World Health Organization Quality of Life 26, *SD* standard deviation.

^a^Skeletal Class I (Japanese normal mean value - 1 SD ≤ ANB angle ≤ Japanese normal mean value + 1 SD), Class II (ANB angle > Japanese normal mean value + 1 SD), and Class III (ANB angle < Japanese normal mean value - 1 SD).

^b^Fisher's exact test.

^c^Mann–Whitney *U* test.

^d^Kruskal–Wallis test with the Bonferroni test.

*P < 0.05; ** *P* < 0.01; *** *P* < 0.001.

In patients with skeletal Class I, the FMA angle (*P* < 0.001, 1 − *β* = 1.00) and Me deviation (*P* = 0.049, 1 − *β* = 0.45) of the SURG group were significantly greater than those of the NONS group. The SURG group had higher STAI-Trait (*P* = 0.012, 1 − *β* = 0.79) and BDI-II (*P* = 0.007, 1 − *β* = 0.78) scores but lower Psych-QOL scores (*P* = 0.010, 1 − *β* = 0.79) than the NONS group. In patients with skeletal Class II, no significant difference in psychological assessment scores was noted, although age was significantly different (*P* = 0.042, 1 − *β* = 0.38). In patients with skeletal Class III pattern, the Psych-QOL score of the SURG group, which had significantly larger SNB (*P* < 0.001) and smaller ANB angles (*P* < 0.001), was significantly higher than that of the NONS group (*P* = 0.047). In each group of orthodontic treatment (NONS and SURG groups), when all values were compared with anterior–posterior skeletal pattern (skeletal Class I, II, and III groups), no significant difference in psychological assessment scores was noted, although some cephalometric analysis values were significantly different.

## Discussion

In this study, patients who chose surgical orthodontic treatment and those with skeletal Class III had higher anxiety and depression scores and a lower body image score. However, based on the LMM, only the choice of surgical orthodontic treatment was the main factor affecting anxiety, depression, and body image scores. Additionally, among the orthodontic patients with skeletal Class I, patients who chose surgical orthodontic treatment show higher anxiety and depression scores or lower body image score, respectively. Among the orthodontic patients with Class III, patients who chose surgical orthodontic treatment show lower body image score.

In this study, three aspects of psychological well-being were evaluated using the STAI, BDI-II, and QOL questionnaires. Japanese versions of these questionnaires have been developed, and their usefulness in screening anxiety, depression, and QOL levels in the general population and orthodontic patients has been reported^13,16^. The BDI-II score has been used in patients aged > 13 years, whereas the STAI and QOL have been used in patients aged > 18 years. However, orthodontic patients aged < 18 years often select treatment methods for final occlusion and facial morphology. It is important to respect children's autonomy and their ability to make treatment decisions for themselves. Some studies have concluded that children aged 14 or 15 years are as competent as adults^17,18^. Typically, in surgical orthodontic treatment, pre-surgical orthodontic treatment is initiated after the adolescent growth spurt, and the surgery is usually planned after the skeletal growth has stabilized at age 17–18 years^19,20^. In this study, the youngest age of patients who planned orthodontic treatment and chose surgical orthodontics was 14 years. Therefore, patients aged ≥ 14 were selected as study participants.

Skeletal pattern and the degree of malocclusion affect the psychological status of orthodontic patients^1–7^. Thus, this study aimed to evaluate how these factors influence anxiety, depression, and body image of orthodontic patients undergoing edgewise treatment. We compared the psychological assessment findings of the patients diagnosed with jaw deformity who chose surgical orthodontic treatment and those who chose edgewise treatment with or without a diagnosis of jaw deformity. It was difficult to classify participants on the basis of jaw deformity diagnoses because of the borderline cases of orthognathic surgery. In particular, diagnosing the indications for orthognathic surgery in patients with skeletal Class II proved challenging because camouflage treatment was easier to perform in these patients than in skeletal Class III patients^21^. Although orthodontists diagnosed jaw deformity, patients with skeletal Class II often chose camouflage treatment. In psychological research, it is very important for patients to wish for and choose the treatment method. Thus, in this study, only patients who were diagnosed as showing jaw deformities by orthodontists and chose orthognathic surgery were defined as surgical orthodontic patients.

In a comparison of the scores of each psychological evaluation for each factor, patients who chose surgical orthodontic treatment and those with skeletal Class III had higher scores for anxiety and depression and a lower score for body image. However, based on the LMM, only the choice of surgical orthodontic treatment significantly affected psychological well-being (*P* < 0.01). As we did not find any significant interaction effect between the choice of surgical orthodontic treatment and the skeletal pattern by ANB angle, our results indicate that the choice of surgical orthodontic treatment affects psychological assessment scores, regardless of the skeletal pattern by ANB angle.

We compared the psychological scores between the groups based on the choice of orthodontic treatment for each anterior–posterior skeletal pattern. Patients with skeletal Class I who selected surgical orthodontic treatment had a significantly greater FMA angle and Me deviation than did those who selected non-surgical orthodontic treatment, and had higher anxiety and depression and lower body image scores than did those who chose non-surgical orthodontic treatment. However, our sample size was very small; thus, we performed post-hoc power calculations (1 − β). Generally, power calculation (1 − β) should be at least 0.8^22^. Our power analysis showed a power of approximately 0.8, except for the measurement of Me deviation. Thus, our results are reliable, despite the small sample size. In patients with skeletal Class II, obtaining statistically reliable results was difficult, as the number of patients who chose surgical orthodontic treatment was very small. A previous study showed that patients with skeletal Class III and jaw deformity had higher scores for depression, hysteria, and psychasthenia^5^. Patients with skeletal Class III who selected surgical orthodontic treatment had a significantly more pronounced mandibular protrusion and lower body image scores than those who selected non-surgical orthodontic treatment. Thus, our results suggest that patients with skeletal Class I or III who were diagnosed with jaw deformities and chose surgical orthodontic treatment may be at a very high risk of developing psychological problems.

It has been reported that the patients who perceive themselves as having a great need for orthodontic treatment are those who have a poor self-perception of oral aesthetics and low self-esteem^23^. Patients with severe malocclusion have poor oral aesthetic self-perception, with aesthetic impact expressed by the constraint on smiling or showing teeth^24^. Malocclusion adversely affects the attractiveness of the face and smile, especially in the case of Class III malocclusion^5–7,25^. Thus, it is known that self-evaluation of facial attractiveness such as smile is closely related to facial morphology and malocclusion. Although this study could not directly show a relationship between facial and oral attractiveness such as self-assessment of smiles and psychological problems, we think that the poor body image shown in the patients with severe malocclusion may be associated with low self-assessment of their facial features.

In clinical practice, psychological assessments by STAI, BDI-II, and QOL are evaluated using cutoff values. Studies examining the proportion of participants whose psychological scale scores are above the normal range can show the proportion of patients with clinical psychological problems requiring counseling^26–28^. However, there is a risk with this method; patients with borderline scores and those with significantly low scores would be considered to have similar psychological states. Thus, we evaluated the psychological status using the total score. In many of the results of this study, when anxiety and depression scores were significantly higher, the body image score was significantly lower. Previous studies in other medical fields have reported that anxiety and depression influence the QOL of patients^29–31^. Our results suggest that the higher levels of anxiety or depression of orthodontic patients may be related to their body image.

## Limitations

This study had some limitations. Firstly, some participants who opted for the non-surgical orthodontic treatment were diagnosed with jaw deformities. As our sample size was very small, the relationship between orthodontic treatment choice and the diagnosis of jaw deformity in each skeletal pattern could not be evaluated. Secondly, the number of patients with skeletal Class I and II who underwent surgical orthodontic treatment was very small. Thirdly, the psychological state was evaluated before the start of edgewise treatment only, and changes after the treatment could not be evaluated. Thus, to understand the psychological state of orthodontic patients in more detail, it is necessary to increase the sample size and investigate the psychological changes after the treatment. Finally, since all adolescents in this study chose orthodontic treatment with their parents present, parental psychology may have influenced the choice of orthodontic treatment for the adolescents. It has been reported that Individuals are judged for their social and intellectual abilities by the appearance and attractiveness of their faces, such as the appearance of their mouth and teeth^32,33^, and attractive children are subsequently treated more positively than less attractive children^34^. Thus, parents think that it is important to have good dentition and a nice smile from the viewpoint of making a good first impression for the future of the child, especially in college and job interviews^35^. However, it was not possible to show how much parental psychology affected the results. In a study of psychological evaluation for adolescent patients, the necessity of a parental psychological survey was pointed out^35^, so it is necessary to consider this in future research.

## Conclusions

In this study, patients who chose surgical orthodontic treatment and patients with skeletal Class III had a higher tendency to show anxiety and depression and a more dissatisfied body image. Moreover, the choice of surgical orthodontic treatment had a significant effect on these psychological states compared with anterior–posterior skeletal pattern. Particularly the patients with skeletal Class I and III, who chose surgical orthodontic treatment, may be more prone to anxiety and depression and/or exhibit more dissatisfied body image.

## Data Availability

The datasets generated during and/or analyzed during the current study are available from the corresponding author on reasonable request.

## References

[1] Dimberg L, Arnrup K, Bondemark L (2015). The Impact of malocclusion on the quality of life among children and adolescents: A systematic review of quantitative studies. Eur. J. Orthod..

[2] Ashari A, Mohamed AM (2016). Relationship of the dental aesthetic index to the oral health-related quality of life. Angle Orthod..

[3] Stagles C, Popat H, Rogers S (2016). Factors influencing patient-reported quality of life in pretreatment orthognathic surgery patients. Angle Orthod..

[4] Burden DJ (2010). Psychological status of patients referred for orthognathic correction of skeletal II and III discrepancies. Angle Orthod..

[5] Takatsuji H, Kobayashi T, Kojima T (2015). Effects of orthognathic surgery on psychological status of patients with jaw deformities. Int. J. Oral Maxillofac. Surg..

[6] de Mendes GA, AdasSalibaGarbin C, da Silva Ferraz FW, AdasSaliba T, IsperGarbin AJ (2019). Dentofacial deformities and implications on quality of life: A presurgical multifactorial analysis in patients seeking orthognathic surgical treatment. J. Oral Maxillofac. Surg..

[7] Agırnaslıgıl MO (2019). The changes of self-esteem, sensitivity to criticism, and social appearance anxiety in orthognathic surgery patients: A controlled study. Am. J. Orthod. Dentofac. Orthop..

[8] Volkweis MCC (2021). Psychological symptoms and salivary inflammatory biomarkers in patients with dentofacial deformities: A case-control study. Sci. Rep..

[9] Kaygısız E, Hacıömeroğlu AB, Yüksel S, Mümin M, Tortop T (2021). The influence of abnormalities in the profile and overjet on psychological well-being. Turk. J. Orthod..

[10] Iizuka T, Ishikawa F (1957). Normal standards for various cephalometric analysis in Japanese adults-Japanese male and female adults. J. Jpn. Orthod. Soc..

[11] Sassouni V (1957). Position of the maxillary first permanent molar in the cephalofacial complex. Am. J. Orthod..

[12] Hidano T, Fukuhara M, Iwawaki M, Soga S, Spielberger CD (2000). New STAI manual State-Trait Anxiety Inventory-Form JYZ.

[13] Azuma S (2008). Beneficial effects of orthodontic treatment on quality of life in patients with malocclusion. Tohoku J. Exp. Med..

[14] Kojima M, Furukawa T (2003). Japanese Version of the Beck Depression Inventory.

[15] Tasaki M, Nakane M, Shobou K, Hhhh H (1997). The WHO-QOL26. Health Division of Mental Abuse Protection of Substance.

[16] Abu Alhaija ESJ, Al-Nimri KS, Al-Khateeb SN (2005). Self-perception of malocclusion among north Jordanian school children. Eur. J. Orthod..

[17] Grootens-Wiegers P, Hein IM, van den Broek JM, de Vries MC (2017). Medical decision-making in children and adolescents: Developmental and neuroscientific aspects. BMC Pediatr..

[18] Weithorn LA, Campbell SB (1982). The competency of children and adolescents to make informed treatment decisions. Child Dev..

[19] McCance AM, Moss JP, Fright WR, James DR, Linney AD (1992). A three dimensional analysis of soft and hard tissue changes following bimaxillary orthognathic surgery in skeletal III patients. Br. J. Oral Maxillofac. Surg..

[20] Suen KS, Lai Y, Ho SMY, Cheung LK, Choi WS (2018). A longitudinal evaluation of psychosocial changes throughout orthognathic surgery. PLoS ONE.

[21] Jung MH (2016). Quality of life and self-esteem of female orthognathic surgery patients. J. Oral Maxillofac. Surg..

[22] Kazis LE, Anderson JJ, Meenan RF (1989). Effect sizes for interpreting changes in health status. Med. Care..

[23] Badran SA (2010). The effect of malocclusion and self-perceived aesthetics on the self-esteem of a sample of Jordanian adolescents. Eur. J. Orthod..

[24] Marques LS, Barbosa CC, Ramos-Jorge ML, Pordeus IA, Paiva SM (2005). Malocclusion prevalence and orthodontic treatment need in 10–14-year-old schoolchildren in Belo Horizonte, Minas Gerais State, Brazil: a psychosocial focus. Cad. Saude Publica..

[25] Reis GM (2021). Smile attractiveness in class III patients after orthodontic camouflage or orthognathic surgery. Clin. Oral Investig..

[26] Hocaoglu M, Ayaz R, Gunay T, Akin E, Turgut A, Karateke A (2020). Anxiety and post-traumatic stress disorder symptoms in pregnant women during the COVID-19 pandemic's delay phase. Psychiatr. Danub..

[27] Westhoff-Bleck M (2020). Diagnostic evaluation of the hospital depression scale (HADS) and the Beck depression inventory II (BDI-II) in adults with congenital heart disease using a structured clinical interview: Impact of depression severity. Eur. J. Prev. Cardiol..

[28] Bi JL (2019). The development and evaluation of a sub-health self-rating scale for university students in China. BMC Public Health.

[29] Li Q, Lin Y, Qiu Y, Gao B, Xu Y (2014). The assessment of health-related quality of life and related factors in Chinese elderly patients undergoing chemotherapy for advanced cancer: A cross-sectional study. Eur. J. Oncol. Nurs..

[30] Saevarsdottir T, Fridriksdottir N, Gunnarsdottir S (2010). Quality of life and symptoms of anxiety and depression of patients receiving cancer chemotherapy: Longitudinal study. Cancer Nurs..

[31] Kato T, Sampei M, Saito K, Morisaki N, Urayama KY (2021). Depressive symptoms, anxiety, and quality of life of Japanese women at initiation of ART treatment. Sci. Rep..

[32] Eagly AH, Ashmore RD, Makhijani MG, Longo LC (1991). What is beautiful is good, but…: A meta-analytic review of research on the physical attractiveness stereotype. Psychol. Bull..

[33] Newton JT, Prabhu N, Robinson PG (2003). The impact of dental appearance on the appraisal of personal characteristics. Int. J. Prosthodont..

[34] Langlois JH (2000). Maxims or myths of beauty? A meta-analytic and theoretical review. Psychol. Bull..

[35] Shah R, AlQuraini N, Cunningham SJ (2019). Parents' perceptions of outcomes of orthodontic treatment in adolescent patients: A qualitative study. Eur. J. Orthod..

